# School Lunch Take up and Attainment in Primary and Secondary Schools in England

**DOI:** 10.3389/fpubh.2015.00230

**Published:** 2015-10-12

**Authors:** Michael Nelson, Karen Gibson, Jo Nicholas

**Affiliations:** ^1^Public Health Nutrition Research Ltd., London, UK; ^2^London Borough of Ealing, London, UK; ^3^Children’s Food Trust, Sheffield, UK

**Keywords:** school lunch, academic attainment, primary, secondary, England

## Abstract

**Hypothesis:**

Average levels of attainment in primary and secondary schools in England in 2010 and 2011 are positively associated with changes in average school lunch take up between 2008–2009 and 2010–2011.

**Subjects/methods:**

Average school lunch take up and attainment data were available for 2009–2011 for primary and secondary sectors in a minimum of 106 local authorities (LAs) in England and 853 individual primary schools in six LAs. Associations between attainment at 11–12 years (primary) and 15–16 years (secondary) and changes in school lunch take up were tested using multilevel analysis, multiple regression, and cross-tabulation (chi-squared analysis).

**Results:**

At school level, attainment at 11–12 years in 2010 and 2011 showed 9 positive and 12 negative associations with changes in school lunch take up between 2009 and 2011. At LA level, average attainment at 15–16 years in 2011 was associated with changes in total school lunch take up in 2010–2011 (*p* = 0.034). Cross-tabulation of changes in attainment 2010–2011 (above or below median) were positively associated with changes in total school lunch take up between 2009 and 2011, by quartiles (Chi-squared = 11.041, df = 3, *p* = 0.012).

**Conclusion:**

Attainment at secondary level in England is statistically significantly associated with increases in healthier school lunch take up. Results in the primary sector are not consistent.

## Introduction

Between 2006 and 2009, compulsory standards for school food were introduced in primary and secondary schools in England (1, 2). From 2005 to 2012, the Children’s Food Trust monitored changes in average school lunch take up (3) in up to 152 local authorities (LAs) in England. It also assessed lunchtime food provision and consumption and nutrient intake of children in primary (4) and secondary schools (5). The Trust also obtained annual data on take up for 2008–2009, 2009–2010, and 2010–2011 from 1190 individual primary schools in 5 LAs, to which Public Health Nutrition Research added data from 55 schools in a sixth LA over the same time period. The introduction of school food standards resulted in an overall improvement in the nutritional quality of the food being provided and consumed at lunch time (4, 5) between 2005 and 2011. Average school lunch take up increased nationally, but decreased in some schools and LAs.

An international review in 2006 (6) suggested that, across a wide spectrum of nutrition, better nutrition is associated with better learning outcomes. In the UK, studies in primary (7) and secondary (8) schools showed that healthier eating at lunchtime was associated with better learning behaviors in the classroom after lunch. Studies by Micha et al. on healthier breakfast in teenagers (9, 10) showed that healthier breakfasts were associated with cognitive function likely to be associated with better learning.

A study by the Institute for Social and Economic Research suggested that improvements in school lunch provision in Greenwich (following the celebrity chef Jamie Oliver’s drive to improve school meals) was associated with higher Key Stage 2 (KS2) results compared with matched neighboring boroughs (11, 12), echoing earlier findings that poor diet in early childhood was associated with lower KS1 and KS2 results (13).

The government’s free school meal (FSM) pilot program showed better attainment among those pupils who had taken up their FSM entitlement (14). Similarly, a review of the impact of participation in the United States subsidized school lunch program suggests that better attainment was a key outcome (15).

If the reported associations between healthier eating and better attainment cited above are valid, then it would be reasonable to assume that, on average, the LAs and schools in which take up had increased between 2009 and 2011 would show better average attainment amongst their pupils compared with LAs and schools where take up had stayed the same or decreased over the same period, and that the greater the increase in take up, the greater the impact on attainment.

### Hypotheses

The present paper explores relationships between levels of attainment in the academic years 2008–2009, 2009–2010, and 2010–2011^1^ and changes in school lunch take up over the same time periods, using data available at either LA level or school level. The hypothesis being tested is

Average levels of attainment at ages 11-12 (Key Stage 2) and 15-16 (Key Stage 4 (GSCE)) in 2010 and 2011 in schools and local authorities in England are positively associated with changes in average school lunch take up (paid for, free, or total) between 2008-2009 and 2010-2011.

## Materials (Or Subjects) and Methods

### Data

School lunch take up data (the percentage of enrolled pupils taking a school lunch, whether paid for, free, or total, primary and secondary level) were collected using a standardized approach in all schools and LAs across England between 2009 and 2011 (16–18). The standardized method required all schools and LAs to report for each academic year the numbers of children on role in primary schools and secondary schools in their jurisdiction, and the number of school lunches provided (paid for, free, and total) in each sector (primary or secondary). The average percentage take up (the number of meals served divided by the total number of pupils enrolled at each school or across all schools in the LA) was calculated for paid for meals, for free meals, and for all meals served (total), separately for primary schools and secondary schools.

In the primary sector, average take up data were available in 130 (86%) of 152 LAs in England across the 2008–2009, 2009–2010, and 2010–2011 academic years. In the secondary sector, average take up data were available at LA level for between 106 (70%) and 119 (78%) of 152 LAs in England across the same three time periods.

At school level, the Children’s Food Trust obtained information on take up in 2008–2009, 2009–2010, and 2010–2011 from 1190 individual primary schools in five LAs, to which Public Health Nutrition Research added data from 55 schools in a sixth LA covering the same time period to make a total of 1245 schools in six LAs, covering 98% of all schools in the LAs (*n* = 1275), and 7.4% of all state funded primary schools in England (*n* = 16,884 in January 2011). The calculation of average school lunch take up (paid for, free, and total) followed the same method as used at LA level.

Average attainment data for primary schools were obtained at LA level for KS2 English and Maths (percentage of pupils achieving Level 4 and above and Level 5 and above in each subject) (19). “Level 4” and “Level 5” represent levels of academic attainment for 11- to 12-year-old children as defined by the Department for Education (20). Average attainment data for secondary schools were obtained at LA level for KS4 (percentage of pupils achieving 5 or more A*–C grades at GCSE^2^ for all subjects, and percentage of pupils achieving 5 or more A*–C grades at GCSE, including English and Maths) from the Department for Education website (19). For the 1245 individual primary schools for which take up data were obtained, average attainment data at school level for KS2 for 2009, 2010, and 2011 were obtained from the Department for Education website (19).

The primary sector analyses were restricted to those LAs and schools in which changes in average total school lunch take up were <20% between the years over which change was being assessed, and in which changes in KS2 results did not exceed 50%. In the primary sector, of the 130 LAs for which data were available, 106 (82%) met these criteria and provided data across all 3 years, and for which data on attainment were available^3^. Of the 1245 schools for which data were available, 853 met the criteria and provided data across all 3 years. These exclusions ensured that the datasets were consistent across all 3 years and avoided extremes of change having an undue influence on the multilevel and regression analyses.

### Statistical Analysis

The impact of changes in school lunch take up on attainment at LA level were assessed using multiple regression in which all predictors were forced to enter the model. The models for analysis for the primary sector are shown in Table 1. For each model, the dependent variable was the percentage of pupils achieving the observed level of attainment (separate analyses were conducted for Level 4 and above and for Level 5 and above, for English and Maths, for 2010 and 2011). Predictor variables included the corresponding levels of attainment for each subject and level in English and Maths in the preceding years (2009 or 2010), the percentage school lunch take up and the change in school lunch take up (separate analyses for paid for, free, and total school meals) in the 1 or 2 years prior to the year of the attainment data (2009–2011 and 2010–2011 for the 2011 attainment data, and 2009–2010 for the 2010 attainment data) and potential confounders of attainment such as Index of Multiple Deprivation (IMD) (21), percentage absenteeism, and percentage of pupils with English as an additional language in the relevant year (2010 or 2011) (19). To take into account differing numbers of primary schools in different LAs, data were weighted according to the number of primary schools in each LA.

**Table 1:** **Multiple regression models for primary sector local authority level analyses**.

Dependent variable		Independent variables
Key stage 2 (% attaining) Separate analyses for English and Maths	Year	Separate analyses for percentage take up of paid for, free, and total school lunches
Level 4 and above	2011	Level 4 and above 2009, take up 2009, change in take up 2009 to 2011, IMD 2011, absenteeism (%) 2011; English as an additional language (%) 2011
Level 4 and above	2011	Level 4 and above 2010, take up 2010, change in take up 2010 to 2011, IMD 2011, absenteeism (%) 2011; English as an additional language (%) 2011
Level 4 and above	2010	Level 4 and above 2009, take up 2009, change in take up 2009 to 2010, IMD 2010, absenteeism (%) 2010; English as an additional language (%) 2010
Level 5 and above	2011	Level 5 and above 2009, take up 2009, change in take up 2009 to 2011, IMD 2011, absenteeism (%) 2011; English as an additional language (%) 2011
Level 5 and above	2011	Level 5 and above 2010, take up 2010, change in take up 2010 to 2011, IMD 2011, absenteeism (%) 2011; English as an additional language (%) 2011
Level 5 and above	2010	Level 5 and above 2009, take up 2009, change in take up 2009 to 2010, IMD 2010, absenteeism (%) 2010; English as an additional language (%) 2010

Similar models were used for analyses of secondary sector data at LA level using KS4 results. Separate analyses were carried out for the percentage of pupils achieving 5 or more A*–C grades at GCSE in any subjects, and 5 or more A*–C grades at GCSE including both English and Maths, for 2011 and 2010, versus changes in the levels of school lunch take up (separate analyses for paid for, free, and total) and for the years shown in Table 1 for the analyses in the primary sector, controlling for attainment and potential confounders (IMD, absenteeism, and English as an additional language) for the corresponding years as shown in Table 1. Data were weighted according the number of secondary schools in each LA.

Local authorities were placed into four equal groups (dividing by quartiles) according to changes in take up (paid for, free, and total), and into two equal groups (dividing by the median) for KS2 and KS4 results. The number of LAs grouped by changes in KS results for 2009–2011 and 2010–2011 were cross-tabulated against grouped changes in take up for both the primary and secondary sectors over the corresponding years. Significance of association was based on chi-squared analysis.

At school level, multilevel models were constructed using MLWiN (22) to assess the impact of school lunch take up and potential confounding factors (as per Table 1) on attainment for English and Maths Level 4 and Level 5, taking into account the clustering of school performance and take up results within LAs. Regression analyses at school level were carried out separately *within* each LA using models with the same structure as set out in Table 1, except that Income Deprivation Affecting Children Index (IDACI) (21) based on school postcode, was used in place of IMD.

### Data Protection and Confidentiality

PHN Research and the Children’s Food Trust work to the data protection principles issued by King’s College London (23) and to the requirements of the Data Protection Act 1998 (24). Information regarding individual primary school take up is kept strictly confidential. All remaining information used in the analyses is in the public domain.

## Results

### Primary Sector

At LA level, the most consistent predictors of KS2 outcomes at Level 4 or Level 5 in 2010 or 2011 (as set out in Table 1) were the corresponding percentage passes at Level 4 or Level 5 in the previous year. Changes in take up (paid for, free, or total) were not predictive of attainment for any of the models based on the LA level data.

The changes in KS2 attainment (above or below median change for 2009–2011, 2009–2010, and 2010–2011) were cross-tabulated against changes in school lunch take up (paid for, FSM, and total) by quartile. None of the cross-tabulations reached statistical significance based on chi-squared analysis.

At school level, using multilevel modeling across all six LAs, the most consistent predictors of KS2 outcomes at Level 4 or Level 5 in 2010 or 2011 were, again, the corresponding percentage passes at Level 4 or Level 5 in the previous year or 2 years. Changes in take up (paid for, free, or total) were not predictive of attainment in any of the models.

To allow for heterogeneity in results between LAs, regression analyses based on the models in Table 1 were carried out separately on school level data within each of the six LAs. The most consistent predictor of KS2 outcomes were, again, the corresponding percentage passes at Level 4 or Level 5 in the previous year or 2 years. There were, however, 21 analyses in which changes in take up were predictive of attainment (Table 2). In LAs 2 and 5, there were positively statistically significant associations between observed levels of KS2 attainment in both 2010 and 2011 at both Level 4 and Level 5 English and Level 4 Maths and increases in school lunch take up (total and FSM). In LA 2, for example, each percentage increase in total school lunch take up between 2009 and 2010 was associated with a 2.4% higher percentage of pupils achieving Level 5 or above in English in 2010 (β = 2.398, *p* = 0.047). In LA 5, a 1% increase in FSM take up between 2010 and 2011 was associated with a 3.7 higher percentage of pupils achieving Level 4 or above in English in 2011 (β = 3.689, *p* = 0.001).

**Table 2:** **Regression coefficients for changes in school lunch take up significantly predictive of Key Stage 2 attainment in the years indicated, selected primary schools^a^, by local authority, England**.

LA	Attainment		Take up		Regression	
	Measure^b^	Year	Source	Years	**β**	*p*
1	English, Level 5	2011	FSM	2009–2011	−1.002	0.010
1	English, Level 5	2010	FSM	2009–2010	−1.492	0.013
1	Maths, Level 4	2011	FSM	2009–2011	−0.893	0.002
2	English, Level 4	2011	Total	2010–2011	1.66	0.018
2	English, Level 5	2011	Total	2010–2011	1.903	0.040
2	English, Level 5	2011	FSM	2009–2010	3.783	0.006
2	English, Level 5	2010	Total	2009–2010	2.398	0.047
2	Maths, Level 4	2011	Total	2010–2011	1.793	0.043
3	English, Level 4	2010	FSM	2009–2010	−0.901	0.024
3	Maths, Level 4	2011	Total	2010–2011	−0.336	0.018
3	Maths, Level 5	2010	FSM	2009–2010	−1.216	0.024
4	English, Level 4	2010	FSM	2009–2010	−2.252	0.031
4	English, Level 5	2011	FSM	2009–2011	−1.169	0.048
4	English, Level 5	2010	Total	2009–2010	−1.109	0.037
4	Maths, Level 4	2010	FSM	2009–2010	−2.789	0.019
4	Maths, Level 5	2011	FSM	2009–2011	−1.437	0.045
4	Maths, Level 5	2010	Total	2009–2010	−1.335	0.028
5	English, Level 4	2011	FSM	2010–2011	3.689	0.001
5	English, Level 4	2011	Total	2010–2011	0.815	0.009
5	Maths, Level 4	2011	FSM	2010–2011	2.176	0.046
5	Maths, Level 4	2011	Total	2010–2011	0.541	0.049

*^a^Numbers in the analyses varied between 36 and 153 schools in individual local authorities, depending on the years, and the numbers of pupils achieving the specified level of achievement*.

*^b^“Level” refers to pupils who had achieved the indicated level or above*.

In contrast, LAs 1, 3, and 4 showed negative associations between school lunch take up and attainment. When explored further, these LAs showed either decreases in levels of attainment over the time period in which school lunch take up was increasing, or variable changes in take up between schools against a backdrop of increasing levels of attainment. There were no statistically significant associations between changes in take up and attainment observed in the sixth LA.

### Secondary Sector

In the secondary sector, using the regression models set out in Table 1 but substituting KS4 results (five or more GCSE passes A*–C, and five or more GCSE passes A*–C including English and Maths) in place of the KS2 results, there were two analyses in which changes in school lunch predicted attainment (Table 3).

**Table 3:** **Regression coefficients for changes in school lunch take up significantly predictive of Key Stage 4 attainment in the years indicated, secondary schools, England**.

Attainment		*n*	Take up		Regression	
Measure	Year		Source	Years	β	*p*
5 or more GCSE A*–C	2011	111	Total	2010–2011	0.080	0.034
5 or more GCSE A*–C including English and Maths	2011	110	FSM	2010–2011	0.083	0.038

Changes in attainment (above or below median change for 2009–2011, 2009–2010, and 2010–2011) were cross-tabulated against changes in school lunch take up (paid for, FSM, and total) by quartile for the corresponding years. There were numerous instances in which the observed cross-tabulations reached statistical significance based on chi-squared analysis, but the trends by quartile were not always consistent. The most consistent statistically significant positive association was for changes in attainment (above or below median change, 2010–2011) cross-tabulated against total school lunch take up 2009–2011 (by quartile), which showed an increase in the percentage of LAs in the upper half of improvement in attainment as the change in total school lunch take up increased (Table 4, Chi-squared = 11.041, df = 3, *p* = 0.012; Mantel–Haenszel test for linear-by-linear association = 10.180, *p* = 0.001). A similar distribution was seen for changes in attainment versus changes in paid for take up (table not shown, Chi-squared = 9.073, df = 3, *p* = 0.028; Mantel–Haenszel test for linear-by-linear association = 7.388, *p* = 0.007).

**Table 4:** Changes in GCSE attainment (5 or more at A*–C) 2010–11 (above or below median) versus changes in average local authority total school lunch take up 2009–11 (by quartile), secondary schools, 106 local authorities, England.

**Change in total school lunch take up, 2009–2011**			**Change in GCSE attainment, 2010–2011**					
			
				**Bottom half**		**Top half**		**All LAs**
			
			Mean	2.30		5.20		3.78
			SD	1.03		1.33		1.89

**Quarter**	**Mean**	**SD**		***n***	**%**	***n***	**%**	

Bottom	−6.3	5.2		19	70.4	8	29.6	27
Second	1.7	1.2		17	60.7	11	39.3	28
Third	4.4	0.6		13	52.0	12	48.0	25
Top	10.0	4.0		7	26.9	19	73.1	26
All LAs	2.4	6.7		56	52.8	50	47.2	106

*Chi-squared = 11.041, df = 3, *p* = 0.012*.

*Mantel–Haenszel test for linear-by-linear association = 10.180, *p* = 0.001*.

## Discussion

The analyses show some positive associations between KS2 and KS4 results in 2010 and 2011 and changes in school lunch take up over the previous years, but overall the results are variable. Moreover, the observed associations may not be causative.

In the primary sector analysis at school level, multilevel modeling showed no association when analyzed across all six LAs. This may be due in part to the heterogeneity across LAs of factors influencing attainment and school lunch take up, especially the timing of the changes in school lunch take up (see end of the Section “Discussion”).

Multiple regression analysis *within* LAs showed positive associations between attainment levels achieved and changes in school lunch take up in two LAs, but negative associations in three others. In most (but not all) of the regression analyses, the observed levels of attainment in 2010 and 2011 were significantly positively associated with the levels of attainment in the previous year. The marker of deprivation IDACI (at school level), the percentage of pupils in the school with English as an additional language, and levels of absenteeism were statistically significantly associated in some of the analyses with the observed levels of attainment, but the associations were not consistent from one LA to the next.

Nine of the 12 negative associations observed in relation to levels of attainment were with changes in FSM take up (the remaining three were in relation to changes in total take up) (Table 2). This may reflect increases in the numbers of pupils from low-income households, where attainment is typically lower (25). In contrast, in LAs 2 and 5, six of the nine positive associations observed were between levels of achievement and changes in total take up (paid for plus free). The size of the influx of pupils from poorer households, the association between poverty and poor academic performance (25), and LAs’ strategies for addressing educational issues in children from low-income families may help to explain why the observed associations between achieved levels of attainment and changes in take up were positive in some LAs, and negative in others.

In the LA level analyses, there were no statistically significant associations observed between average levels of KS2 attainment and changes in school lunch take up. Again, issues of timing of changes in take up and heterogeneity across LAs may have mediated against observing associations. The present findings do not accord with those by Belot and James (12) which showed a greater increase in attainment levels (average KS2 score for English and Science) between 2002 and 2007 in one LA (Greenwich, which had taken part in the “Feed Me Better” campaign led by celebrity chef Jamie Oliver) compared with a group of control LAs (26). Analysis of the impact of the introduction of universal FSM in Durham and Newham also showed a positive association between universal FSM provision in primary schools and improvements in KS2 Level 4 results compared with control areas (14).

The small but statistically significant association at LA level between KS4 results and changes in take up (Table 3) suggest that changes in take up were independently associated with attainment even when potential confounders were taken into account. The statistically significant cross-tabulation shown in Table 4 also suggests there was a positive association across LAs between increases in total school lunch take up and being in the upper half of improvements in GCSE results between 2010 and 2011. This is the first time to our knowledge that the potential impact of school food on attainment in secondary schools in England has been demonstrated at LA level.

The data in the present study are derived from a natural ­experiment over time rather than cross-sectional analyses of contemporaneous data. This strengthens the argument that the observed changes in school lunch take up may be causally associated with attainment. However, there are numerous potential confounders (in addition to those included in the regression analyses) that may have influenced the observed associations between attainment and changes in school lunch take up, but for which no measure was available.

The most important of these may be the nature and timing of the changes in school lunch take up and compliance with the standards. Changes in catering practices in schools in England began in 2006, immediately after the publication of the interim standards for school food (27), even though compliance with the final standards (1, 2) was not required until September 2008 in the primary sector and September 2009 in the secondary sector. Caterers reported finding it easier to make changes in primary schools than in secondary schools, both to increase take up and to make school food compliant with the standards: engagement with pupils and parents was more straightforward; younger pupils were more amenable to the changes being introduced, and the scale of change in a primary school was easier to manage (3, 28, 29). Thus, if associations between attainment and changes in take up were most evident during the initial increases in take up, which began in the primary sector in 2007–2008, they may have preceded the period being investigated in the present study. The later introduction of changes in the secondary sector might help to explain why statistically significant associations between attainment and changes in school lunch take up at LA level were observed in the secondary sector but not in the primary sector.

Other factors may have contributed to the observed positive associations. A head teacher interested in improving school lunch take up (and undertaking the necessary steps to bring that about) may also have been undertaking other activities within the school to improve attainment, for example, improving the dining environment and facilitating better pupil interactions; helping pupils to attain lunchtime aspirations (30), thereby helping to increase concentration in the period immediately after lunch; (7, 8) strengthening the role of pupil-led School Councils; introducing a breakfast club; and introducing and/or enforcing stay-on-site policies in secondary schools. Changes in school lunch take up may therefore have been a marker for other changes in the school environment associated with improved attainment.

## Conclusion

The present findings on the association between attainment and changes in school lunch take up are variable. Nevertheless, they contribute to a growing body of evidence which suggests that, in the right circumstances, higher levels of consumption of healthier food in schools can promote higher levels of attainment at both primary and secondary level.

## Conflict of Interest Statement

The authors declare that the research was conducted in the absence of any commercial or financial relationships that could be construed as a potential conflict of interest.

## Funding

The research was funded by the Children’s Food Trust and the London Borough of Ealing.

## Footnotes

^1^For simplicity of expression, the changes in take up in 2008–2009 and measures of attainment for that academic year are referred to as 2009; similarly, “2010” covers the 2009–2010 academic year, “2011” the 2010–2011 academic year.

^2^GCSE – General Certificate of Secondary Education. See https://www.gov.uk/national-curriculum/key-stage-3-and-4

^3^Some values for Key Stage results are not published by the Department for Education when there is concern about the representativeness of the findings or the risk of being able to identify individual schools or pupils.
